# Surface-coated magnetic nanostructured materials for robust bio-catalysis and biomedical applications-A review

**DOI:** 10.1016/j.jare.2021.09.013

**Published:** 2021-10-04

**Authors:** Muhammad Bilal, Hafiz M.N. Iqbal, Syed Farooq Adil, Mohammed Rafi Shaik, Abdelatty Abdelgawad, Mohammad Rafe Hatshan, Mujeeb Khan

**Affiliations:** a School of Life Science and Food Engineering, Huaiyin Institute of Technology, Huaian 223003, China; b Tecnologico de Monterrey, School of Engineering and Sciences, Monterrey 64849, Mexico; c Department of Chemistry, College of Science, King Saud University, P.O. Box 2455, Riyadh 11451, Kingdom of Saudi Arabia; d Department of Industrial Engineering, College of Engineering, King Saud University, P.O. Box 800, Riyadh 11421, Kingdom of Saudi Arabia

**Keywords:** Nanomaterials, Engineered nano-matrices, Enzyme immobilization, Bio-catalysis, Surface functionalization

## Abstract

**Background:**

Enzymes based bio-catalysis has wide range of applications in various chemical and biological processes. Thus, the process of enzymes immobilization on suitable support to obtain highly active and stable bio-catalysts has great potential in industrial applications. Particularly, surface-modified magnetic nanomaterials have garnered a special interest as versatile platforms for biomolecules/enzyme immobilization.

**Aim of review:**

This review spotlights recent progress in the immobilization of various enzymes onto surface-coated multifunctional magnetic nanostructured materials and their derived nano-constructs for multiple applications. Conclusive remarks, technical challenges, and insightful opinions on this field of research which are helpful to expand the application prospects of these materials are also given with suitable examples.

**Key scientific concepts of review:**

Nanostructured materials, including surface-coated magnetic nanoparticles have recently gained immense significance as suitable support materials for enzyme immobilization, due to their large surface area, unique functionalities, and high chemical and mechanical stability. Besides, magnetic nanoparticles are less expensive and offers great potential in industrial applications due to their easy recovery and separation form their enzyme conjugates with an external magnetic field. Magnetic nanoparticles based biocatalytic systems offer a wide-working temperature, pH range, increased storage and thermal stabilities. So far, several studies have documented the application of a variety of surface modification and functionalization techniques to circumvent the aggregation and oxidation of magnetic nanoparticles. Surface engineering of magnetic nanoparticles (MNPs) helps to improve the dispersion stability, enhance mechanical and physicochemical properties, upgrade the surface activity and also increases enzyme immobilization capabilities and biocompatibility of the materials. However, several challenges still need to be addressed, such as controlled synthesis of MNPs and clinical aspects of these materials require consistent research from multidisciplinary scientists to realize its practical applications.

## Highlights

• Explores Applications of Enzymatic catalysis.
• Enzymes destabilize in harsh conditions which increase their bioprocess cost.
• Enzyme immobilization enhances the biocatalytic performance of enzymes.
• Nanomaterials are intriguing supporting matrices for enzyme immobilization.
• Discussed enzyme immobilization on multifunctional magnetic nanomaterials (MNPs).
• Biomedical applications and future prospects of enzyme coated MNPs are summed up.

## Graphical abstract

**Figure:**
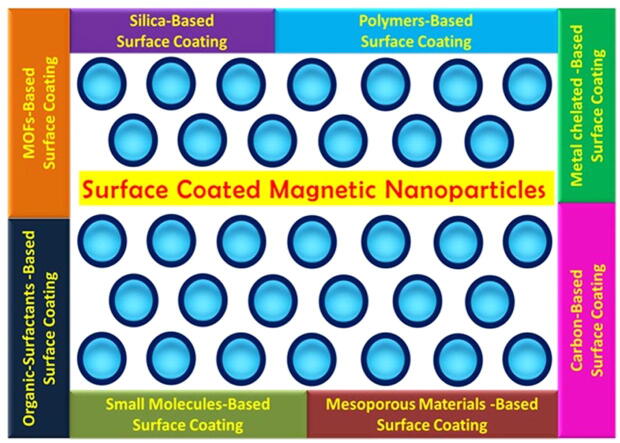

## Introduction

Magnetic nanoparticles (MNPs) have recently emerged as fascinating nanomaterials that have garnered extensive research attention among the scientific community and researchers owing to their broad-spectrum applications in numerous fields including nano-biomedicine [1], environmental protection [2], catalysis [3], electronic communication [4], magnetic fluids [5], data storage [6], etc. In addition to some special magnetic features, i.e., superparamagnetism, magnetic nanomaterials also exhibit exceptional physical attributes, stability, and biocompatibility [7]. Amongst various kinds of MNPs, including iron oxides (Fe_3_O_4_ and γ-Fe_2_O_3_), alloy-based (CoPt_3_ and FePt), pure metal (Fe and Co), and spinel-type ferromagnet (MgFe_2_O_4_, MnFe_2_O_4_, and CoFe_2_O_4_) MNPs, magnetite iron oxide (Fe_3_O_4_) nanoparticles are of paramount importance and gained popularity in biomedicine and biocatalysis due to facile fabrication, low toxicity, and acquiescence to surface functionalization [8], [9], [10], [11], [12]. Many literature reports have revealed the frequent use of magnetic Fe_3_O_4_ nanoparticles as a drug vehicle in cancer theranostics, protein purification, gene delivery, MRI agents, cell labeling, bioseparation, immunoassays, biosensors, and hyperthermia treatment [13], [14], [15], [16], [17].

Different attributes such as morphology, shape, size, and dispersibility of the Fe_3_O_4_ nanoparticles might have an influence on their application in different fields [18]. Thus, researchers have adopted multiple routes to develop MNPs for controlling their shape, size, and morphology with requisite and tailorable features. To date, a large number of fabrication approaches, including microemulsion, co-precipitation, thermal decomposition, hydrothermal, laser pyrolysis, electrochemical deposition, sonochemical and solvothermal methods, microwave-driven method, aerosol pyrolysis, chemical vapor deposition and biobased methods have been proposed for the development of magnetic Fe_3_O_4_ nanoparticles [7]. The biobased synthesis method has been emerged as a promising alternative for obtaining MNPs [19]. These methods are generally executed under the conditions of temperature and pressure close to those of the environment, and utilizing biomolecules extracted from plant extracts or biological entities (e.g., alkaloids, terpenoids, polyphenols, exopolysaccharides, carbohydrates, auxins, flavonoids) [20], [21]. The biobased procedures entail low energy requirements and safer reagents compared to traditional co-precipitation or reduction by agents with adverse impacts. Thus, this biosynthetic approach is recognized as one the most interesting options to advance *trans*-materialization and energy efficiency processes in the nanotechnology perspective [22], [23]. Table 1 depicts notable synthesis procedures along with their merits and demerits. In this report, we elaborate on recent progress in the surface coating strategies and immobilization of various enzymes on the surface-functionalized magnetic nanostructured materials and their derived nanocomposites.

**Table 1:** Synthesis methods for magnetic nanoparticles.

Name of synthesis method	Merits	Demerits
Chemical co-precipitation	Simple and efficient	poor crystallinity, size distribution, and aggregation Not appropriate to prepare highly pure, accurate stoichiometric phase
Microemulsion	Good homogeneous nature Precise control of particle size	time laborious, requirement of large amounts of solvent and poor yield
Hydrothermal reactions	Easy to control particle Shape and size	High pressure, prolonged reaction duration and high reaction temperature
Thermal decomposition	High yield and good control of size and shapes	Elevated reaction temperature
Sol-gel reactions	Good control of size and structure	Prolonged reaction time and cost expensive
Electrochemical method	Easy control of size	Reproducibility
Vapor phase method	Greater yield	High temperatures
Bio-based method	Nontoxic, cost effective, cheap materials and solvents, environmentally friendly, and synthesis at room temperature and atmosphere	Scale up limitations, Reproducibility, tedious purification, and poor understanding of the explicit mechanism.

## Surface modification/functionalization of MNPs for enzyme immobilization

**Fig. 1:**
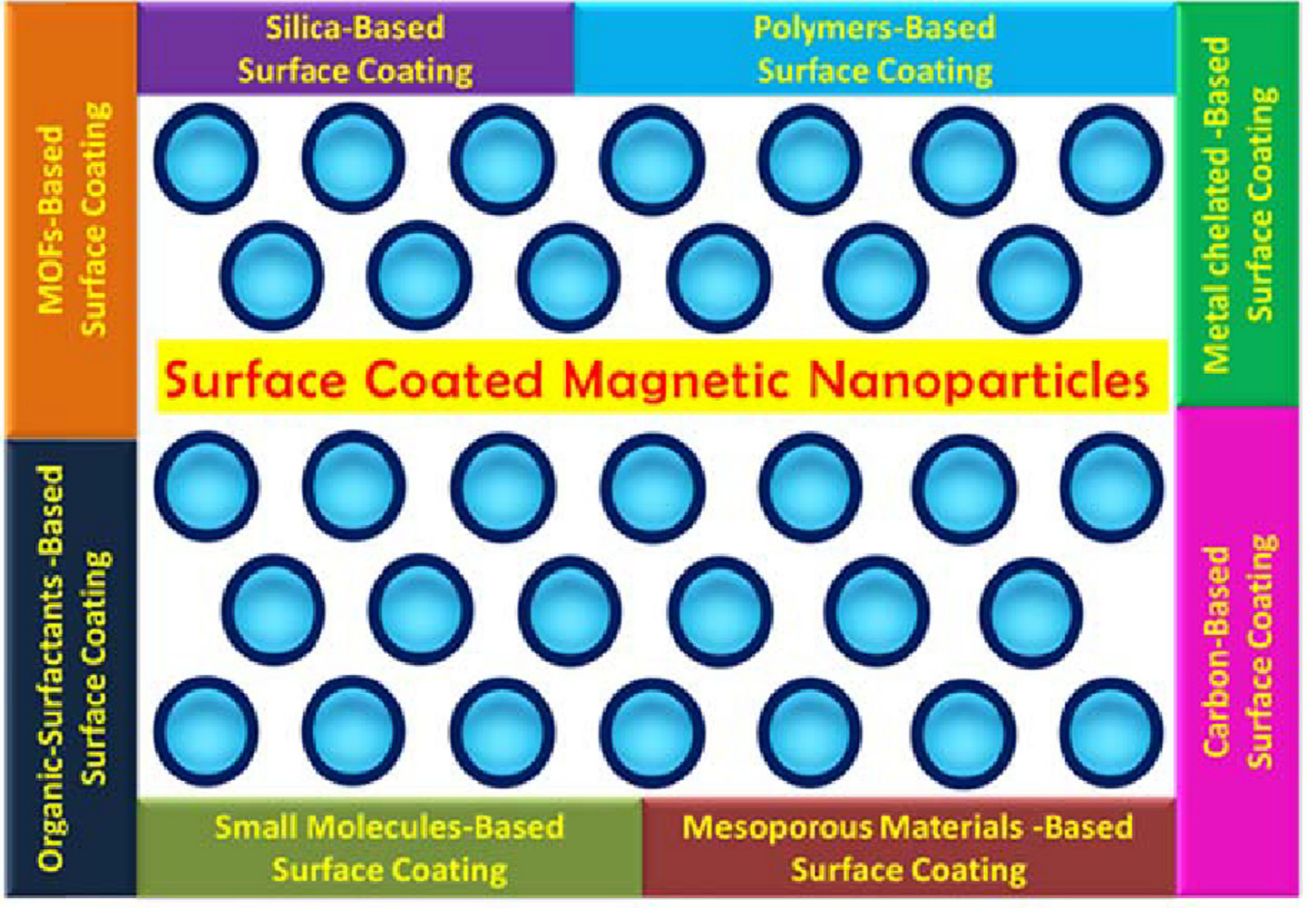
Surface functionalization strategies of MNPs to improve their properties for enzyme immobilization.

**Fig. 2:**
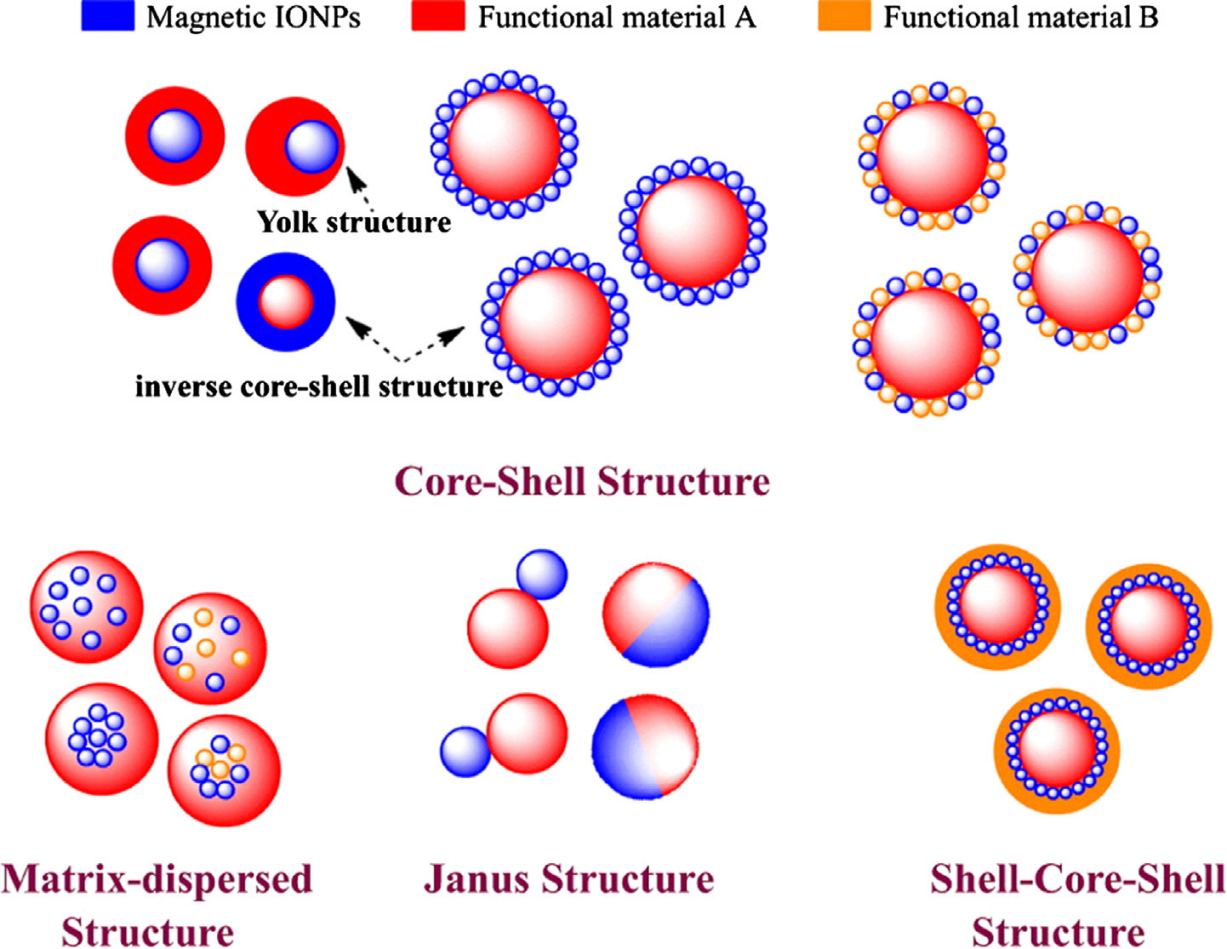
Typical morphologies of magnetic composite nanomaterials. Reprinted from Ref. [26] with permission under the terms of the Creative Commons Attribution 3.0 license. IONPs—iron oxide nanoparticles.

It is demonstrated that Fe_3_O_4_ nanoparticles are prone to aggregate due to their high surface energies, reactivity, and considerably high specific surface area. Moreover, the pristine form of Fe_3_O_4_ NPs exhibits high chemical activity and tend to oxidize in the air resulting in the loss of dispersibility and magnetic properties [24], [25]. Due to these factors, the efficiency of magnetic-driven separation is reduced, and consequently, limited the capability of bare Fe_3_O_4_ NPs for direct immobilization of biomolecules and enzymes. In this avenue, surface modification and functionalization is a necessary step to circumvent the aggregation and oxidation of these nanoparticles. Fig. 1 represents the surface functionalization strategies of MNPs to improve their properties for efficient enzyme immobilization. Four major purposes of surface engineering of MNPs are; 1) improve the dispersion stability of MNPs 2) modify the mechanical and physicochemical properties, 3) upgrade the surface activity of MNPs, and (4) enhance the biocompatibility of MNPs. Taking into account many strategies, researchers have made efforts to fabricate various kinds of magnetic iron oxide nanocomposites as shown in Fig. 2 [26].

## Silica-coated MNPs for enzyme immobilization

Silica coating, in which silica shells are formed on the surface of magnetic cores, is a persuasive technique to functionalize Fe_3_O_4_ NPs. The formation of silica shells is likely to augment the chemical steadiness by providing protection to magnetic cores both from oxidation and aggregation. It can also increase the biocompatibility and hydrophilicity. Insertion of a vast number of silanol groups on the magnetic core’s surface by modification process affords the underlying foundation for additional functionalization with reagents for enzymes attachment. Fe_3_O_4_ NPs can be directly functionalized with silica shells by a well-characterized sol–gel procedure [27], in which tetraethoxysilane is hydrolyzed under alkaline environments to form silica shells on the surface of magnetic cores yielding core–shell Fe_3_O_4_@SiO_2_ NPs. The resultant core–shell Fe_3_O_4_@SiO_2_ NPs are first coated with amino silane [28], or epoxy silane coupling agents [29] to insert amine or epoxy moieties on the surfaces of carrier support, respectively, for effective attachment of enzymes. Afterward, enzymes can be integrated on the surface of the amino group bearing Fe_3_O_4_@SiO_2_ NPs based on Schiff base linkages by using bifunctional reagent glutaraldehyde. Whereas, epoxy-functionalized support matrices can be applied to direct enzyme immobilization via the reaction between epoxy groups of support material and amino groups of enzymes. With reference to amino-functionalized silica-coated magnetite nanoparticles, epoxy-functionalized Fe_3_O_4_@SiO_2_ NPs do not necessitate any additional linkage for enzyme immobilization. However, the immobilization procedure is time-consuming and ineffective due to the slow reactions between epoxy and amino groups, by virtue of steric hindrance associated with the dimensions of the macromolecule [30]. Amino and carboxyl group incorporated MNPs were prepared to gain an insight into whether the basic or acidic modification was more effective for the immobilization of L-asparaginase (ASNase) enzyme. The prepared nanoparticles were characterized by Fourier-transform infrared spectroscopy (FTIR), scanning electron microscopy (SEM), energy-dispersive X-ray spectroscopy (EDAX), X-ray diffraction (XRD), and vibrating-sample magnetometer (VSM). In comparison to the free state of the enzyme, the nanocarriers-supported ASNases were more stable in a broad range of temperature and pH values under the optimum reaction conditions. Likewise, the nanobiocatalysts presented high stability at a raised temperature of 50 °C for 3 h. Free form of the enzyme showed only 30% of its original activity after preserving at 4 °C for 1 month, whereas Fe_3_O_4_/SiO_2_/COOH and Fe_3_O_4_/SiO_2_/NH_2_ ASNase preserved above 56.5% and 78.9% of their preliminary activities, respectively, under identical conditions. Both of the engineered ASNase revealed outstanding functioning stability after 17 consecutive batch cycles [31]. The MNPs surface was functionalized with aminopropyltriethoxysilane (APTES) and polyamidoamine (PAMAM) dendrimer to comprehend the covalent binding of cholesterol oxidase and esterase for the development of cholesterol biosensor [32].

Silica-encapsulated MNPs synthesized by microemulsion techniques were deemed as a good support matrix for immobilizing glucose oxidase (GOD). The binding of GOD onto the support was confirmed by the FTIR spectra. Immobilized bioconjugate preparation maintained over 95% and 90% of its primary activity after storage for 45 days, and 12 consecutive reaction cycles. Substantial improvements in thermal stability profiles were also recorded at high temperatures up to 80 °C. Moreover, the immobilized biocatalyst was less likely to be affected by alterations in pH values [33]. Correa et al. (2020) tested three different types of immobilization methods for covalent bonding of β-Glucuronidases on MNPs and three catalysts for Si particle deposition [34]. Among the nine different immobilized micron-sized biocatalytic preparations, only two showed insignificant activity. All the preparations had superior thermal, storage, and functional stability than the free enzyme. Different bioconjugates with MNPs and Si maintained 40% of their original activities at a high temperature of 80 °C after 6 h, while the free form of enzyme dropped over 90% of its activity within 10 min.

## Polymer-modified MNPs for enzyme immobilization

Generally, ex-situ and in-situ modification are two methods, which are involved in the functionalization of MNPs with organic polymers [35], [36], [37], [38]. The in-situ modification method entails the inclusion of organic polymers in the precursor solution as a stabilizer to generate Fe_3_O_4_ NPs, whereas, in the ex-situ modification approach, the monomers are polymerized on the surface of Fe_3_O_4_ NPs. The generation of effective steric repulsion forces from polymer coatings weakens the magnetic and Van der Waals interactions of Fe_3_O_4_ NPs that subsequently prevent aggregation and augment their stability and dispersibility attributes. In the last few years, a plethora of polymers has been proposed as adequate coating materials to functionalize Fe_3_O_4_ NPs for efficient enzyme immobilization. Many of these polymers have been found suitable agents, such as starch, alginate, albumin, chitosan, polyvinyl alcohol (PVA), polyethyleneimine (PEI), polydopamine, polyethylene glycol (PEG), and different polyoxamines for MNPs development because of their desired biodegradable and biocompatible properties [36], [37], [38], [39], [40], [41]. Chitosan-modified MNPs are largely synthesized by the in-situ modification approach [42]. Dextran is among the choice coating polymers that are used for MNPs functionalization due to non-toxicity, biocompatibility, and degradability by dextranase [43], [44]. It is demonstrated that MNPs coating with dextran can ameliorate their attributes for colloidal stability, and drug delivery, and thus seemed a useful approach in fabricating MNPs [45], [46]. Indeed, dextran is a hydrophilic polymer that is composed of numerous glucose monomers linked together by α-1, 6-glycosidic bonds. This polymer is physically adsorbed onto MNPs by non-covalent linkages in alkaline solutions. The utilization of carboxymethyl dextran (CMD), a derivative of dextran, to fabricate MNPs provides hydroxyl as well as carboxyl functional moieties, which render easy chemical modification. CMD is endowed with excellent biocompatibility, biodegradability, and high-water solubility [47]. Vasić et al. (2020) inspected the influence of different concentrations of CMD on the properties of CMD-wrapped MNPs [48]. The surface morphology and functional groups of CMD-MNPs were monitored by SEM FTIR. The as-synthesized CMD-coated MNPs were then employed as support carriers for immobilizing alcohol dehydrogenase (ADH). Coating of CMD onto MNPs imparts preferable structural and magnetic properties and can thus be applied as a nanocarrier for enzyme immobilization. In contrast to the free form of ADH that dropped 70% of its original activity at 20 °C, and complete loss of its activity at 40 °C after 24 h. The nanoimmobilized biocatalyst retained more than 50%, and 75% of its remaining activity at 20 °C and 40 °C, respectively, under the same incubation period of 24 h.

Francolini et al. (2020) prepared polymer-coated MNPs showing long alkyl chains, either hexadecyl (C16) or octyl (C8) to immobilize *Candida rugosa* lipase (CRL) [49]. Among the nanocarrier supports tested, the one displaying the longest alkyl chains deliver the most promising efficacies for immobilized enzyme. The nanoimmobilized biocatalytic system with the longest alkyl chains also presented superior tolerance to high temperature (ranging from 25 to 70 °C) than the soluble lipase. It also showed good recyclability in four successive cycles and conveniently recovered by a simple magnetic separation. Lipase enzyme from *Thermomyces lanuginosus* (TLL) was covalently attached to PEI-coated new heterobifunctional support, divinyl sulfone (DVS) superparamagnetic nanoparticles (SPMNs) and characterized. PEI is an organic polymer with the highest positive charge density, which provides colloidal stabilization to the protein molecules even at a high concentration of salt [50], [51], [52]. Moreover, as a smart polymer, PEI can respond to various external stimuli like pH, temperature, etc. [53]. Therefore, PEI can be utilized as a multipurpose agent to develop biocatalysts by simple adsorption or multipoint covalent coupling [52]. Its unique chemical structure can confer stabilization to multiple subunits of enzymes and provide stability against various organic solvents. Further, it is used for the co-immobilization of cofactors and enzymes, PEI-functionalized supports for enzyme immobilization might produce inter- and intra-molecular crosslinking, imparting a greater extent of homogeneity of activated amino groups [54]. Thermal inactivation profile at different pH values revealed that the immobilized TLL bioconjugates exhibited the most robust stability at an immobilization pH of 5.0. The nanobiocatalytic preparation obtained at pH 5.0 and blocked with ethanolamine (ETA) and ethylenediamine (EDA) for hydrolyzing racemic methyl mandelate. It achieved excellent enantioselectivity of 72% and 68%, respectively together with greater biocatalytic efficiencies in the reaction system at a neutral pH of 7.0. The engineered system had impressive operational stability retaining over 60% of conversion efficiency after seven repeated cycles.

Chitosan is a linear polysaccharide consisting of glucosamine with varying degrees of deacetylation. It can be utilized for the decoration of the magnetic nanocomposite’s surface. In addition to impart stability to nanocarrier supports, it provides functional groups (–NH_2_ and –OH groups), which are involved in chemical connection with biomolecules. Since the amine groups of chitosan have pKa value near 6.5, it possesses a coiled structure and can be more soluble in acidic solution. A set of desirable characteristics like bio-renewability, hydrophilicity, biodegradability, and biocompatibility [55], [56], the chitosan-induced chemical modifications do not alter the basic network of carrier support and even exert some advantageous activities such as adsorption, chelation, bacteriostatic properties activities [57], [58]. Due to its renewable nature (derived from shells of shellfish: krills, crabs, shrimps, and lobsters, and from the fishing industry waste), chitosan is potentially considered an inexpensive coating agent that additionally play a role in the development of competitive biocatalysts [59]. A novel type of magnetic nanobiocatalyst was designed by efficient immobilization of *Trichoderma reesei* cellulase onto chitosan modified Fe_3_O_4_/graphene oxide nanocomposite (Fe_3_O_4_/GO/CS). Using a covalent coupling method using glutaraldehyde as a cross-linker, cellulase was covalently attached to this nanocomposite. Transmission electron microscopy (TEM), SEM and FTIR confirmed the successful immobilization of cellulase with Fe_3_O_4_/GO/CS. With regard to the soluble enzyme, the nanobiocatalytic system showed highly enhanced bioactivity and retained over 75% of its actual activity. After the immobilization process, a substantial widening in pH, storage, and thermal stability were obtained. The immobilized cellulolytic enzyme was capable of maintaining a high degree of its original activity after repeatedly using for 8 cycles [60]. Covalent attachment of Lipase B from *C. antarctica* onto sebacoyl-modified chitosan-decorated MNPs constitutes a robust nanobiocatalyst to catalyze enzyme-assisted kinetic resolution of various racemic heteroarylethanols. It showed activity up to 10 repeated catalytic cycles under the optimized conditions (n-hexane, vinyl acetate, 45 °C) [61]. Manganese peroxidase isolated from *Anthracophyllum discolor* was immobilized to chitosan/magnetic Fe_3_O_4_ biocomposite to eliminate reactive orange 16 and methylene blue dye pollutants. The nanobioconjugate preparation retained its activity and demonstrated recycling ability in 5 consecutive reaction cycles [62]. Alnadari and coworkers (2020) investigated the comparative use of chitin, chitosan, and sodium alginate as biocompatible polymers to functionalize MNPs for immobilization of β-glucosidase from *T. maritima* (Tm-β-Glu) [63]. This exclusive technology entails a novel thermally resistant chitin-binding domain (Tt-ChBD), which was found desirable for larger-scale applications. Characterization indicated that chitin-coated MNPs exhibited the maximum enzyme loafing capability and galactooligosaccharides (GOS) biosynthesis from lactose among all the immobilization methods for Tm-β-Glu-Tt-ChBD, in comparison to the free form of the enzyme. Chitin represented the most robust binding capacity by combining target proteins with Tt-ChBD. Fascinatingly, magnetic separation enables the reusability of the nanobiocatalytic system in several successive batches for GOS synthesis without a substantial loss of enzyme activity. After the immobilization process, the immobilized enzyme showed operational stability under varying pH, temperature, storage, and thermal conditions.

**Fig. 3:**
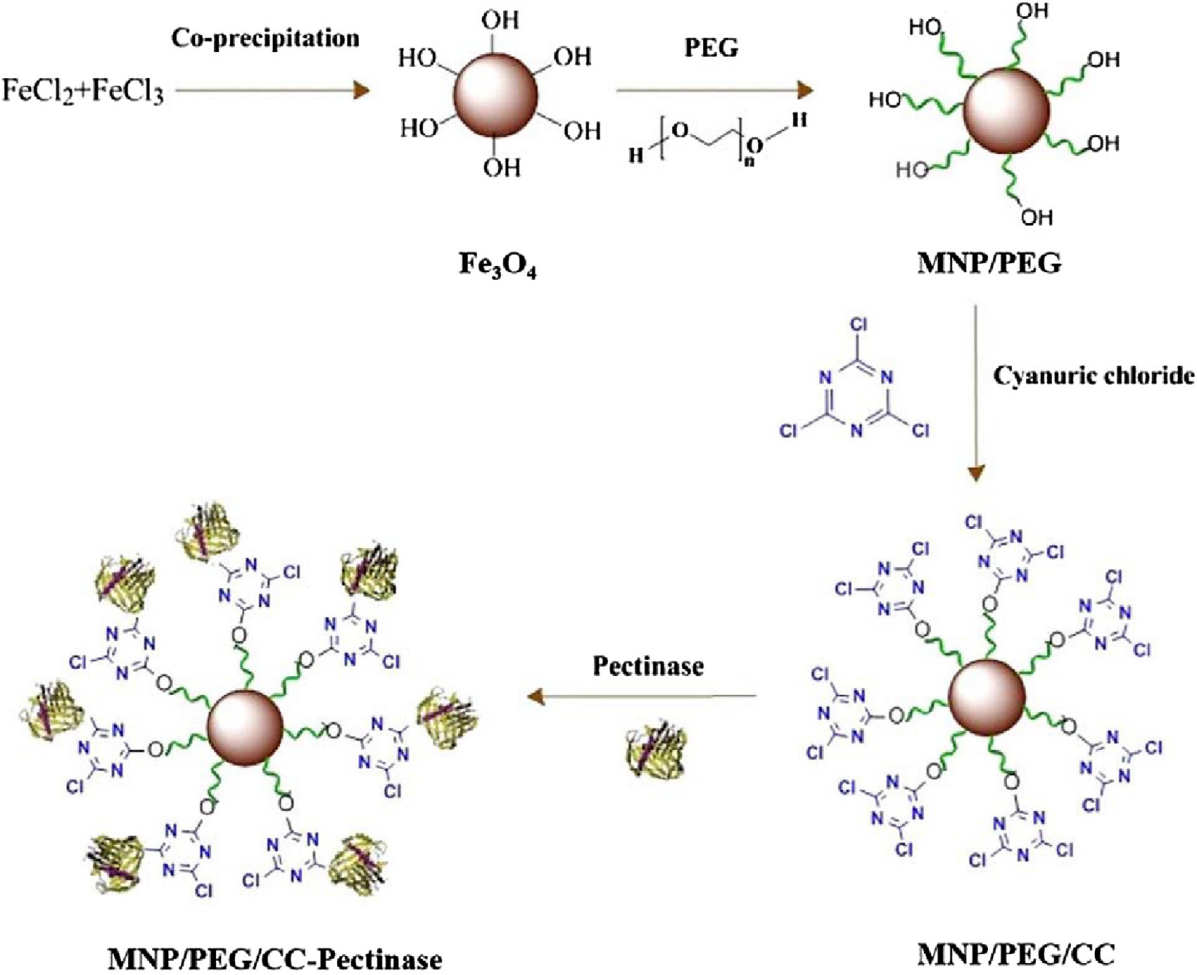
Schematic representation of the synthesis of 1,3,5-triazine-functionalized PEG-coated Fe_3_O_4_ nanoparticles and immobilization of pectinase. Reprinted from Ref. [65] with permission from Elsevier. License Number: 5092320806715.

Polyethylene glycol (PEG) is a hydrophilic polymer of ethylene oxide that can improve the stability of MNPs. Inimitable attributes including water solubility, biocompatibility, flexibility, non-toxicity, and non-antigenic behavior render PEG a suitable coating material in various biotechnological applications. PEG-wrapped MNPs are found to be chemically stable and more dispersible than their pristine form of nanoparticles [64]. In a recent study, PEG-grafted MNPs were applied to covalent immobilization of pectinase via trichlorotriazine crosslinking. The MNPs-PEG were prepared under alkaline conditions by a chemical co-precipitation of FeCl_3_ and FeCl_2_ solutions using PEG as a coating agent. The prepared MNP-PEG nanosupport was then cross-linked with cyanuric chloride to introduce active groups on their surface facilitating the covalent attachment of pectinase enzyme. The resulted modified MNPs were allowed to react with pectinase solution yielding active MNP/PEG/CC-pectinase biocatalytic system (Fig. 3). In addition to high loading capacity, PEG-grafted MNPs immobilized enzyme presented improved satisfactory operational stability, improved catalytic efficiency, and easily recyclability in multiple cycles. pH and thermal stability profile revealed augmented enzyme performance even at extreme values than the free enzyme. Immobilized preparation was able to retain up to 94% and 55% of its actual activity after storage for 125 days at 25 °C, and 10 repeated catalytic runs, respectively. A prominent reduction in turbidity of pineapple juice (up to 59%) after treatment with the immobilized enzyme suggest its application in food-processing sectors [65].

## MNPs and carbon based nanocomposites for enzyme immobilization

MNPs and carbon based nanocomposites that integrates the useful magnetic attributes of the core (high saturation magnetization) and excellent physicochemical properties of carbonaceous materials have been well documented in the scientific literature [66], [67]. The integration of MNPs and carbonaceous materials including CNTs and graphene into nanocomposites has recently become a hot topic of research due to their new and/or enhanced functionalities that cannot be achieved by either component alone, and therefore holds great promise for a wide variety of applications in catalysis, optoeletronic materials, surface enhanced Raman Scattering, biomedical fields, and so on [68]. In comparison to iron oxides, the applications of the carbon-coated MNPs have been revealed in semi-heterogeneous catalysis, electrode supercapacitors, lithium-ion batteries, and blood and water purification [69], [70], [71]. Uniform coating of carbon on the surface of MNPs could protect from air oxidation and increase its stability, biocompatibility, and dispersible properties. For biocatalysis application, the aptitude of enzyme connection with the nanosupport by an adequate organic chemistry-based protocol is particularly alluring because it enables better enzyme reusability and negligible protein side-products in the final reaction mixture. Owing to high magnetic saturation, larger surface area, and tunable surface functionalities, carbon-coated MNPs have emerged as attractive choices of nanosupports for enzyme immobilization. Zlateski et al. have used chemically functionalized (diazonium chemistry) carbon-coated cobalt NPs, which is activated for bioconjugation (N,N-disuccinimidyl carbonate) and used for enzyme immobilization [72]. Three different kinds of enzymes including α-chymotrypsin, lipase B, and β-glucosidase were covalently attached to this magnetic nanosupport. After immobilization, the resultant enzyme–particle conjugates showed good stability and catalytic performance and could be recyclable from milliliter to liter volumes in short recycling durations. Magnetic carbon-coated NPs have been used for horseradish peroxidase (HRP) immobilization in combination with chitosan and cross-linking of glutaraldehyde and applied to constitute an enzyme-based novel amperometric electrode for H_2_O_2_ sensing [73].

**Fig. 4:**
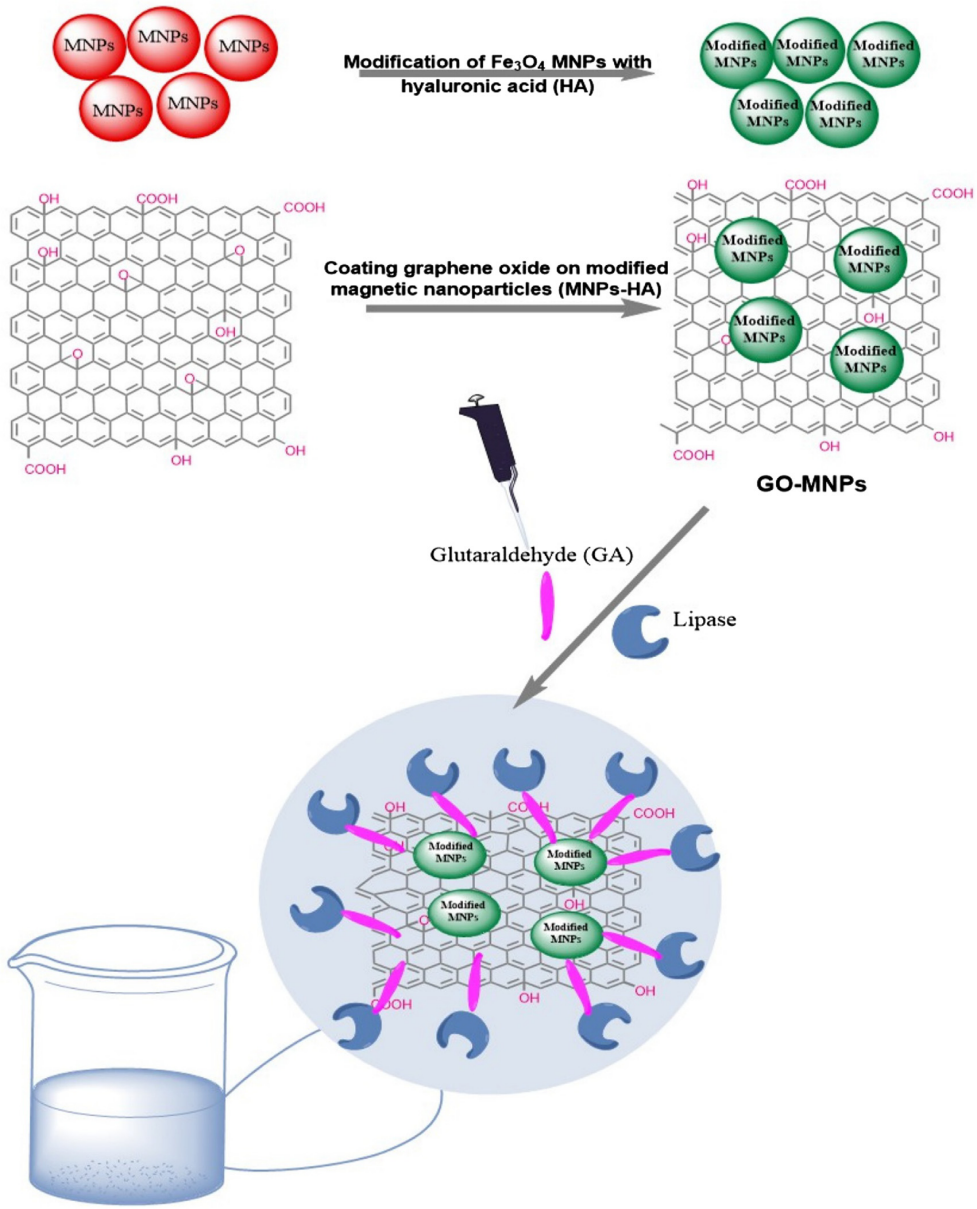
The diagram of the lipase-GO-MNPs-CLEAs assembly process. Reprinted from Ref. [82] with permission from Elsevier. License Number: 5092320991917. CLEAs-cross-linked enzyme aggregates.

Carbon nanotubes (CNTs) are one-dimensional nanostructured materials, which are prepared by the coiling of one or more layers of graphite sheets around a central axis. Many studies have focused on functionalization (or coating) of CNTs (i.e., with magnetic or superparamagnetic nanoparticles) or filling their cavity with magnetic molecules to obtain versatile systems for biomedical applications [74]. CNTs have recently drawn much interest in the fabrication of many nanocomposites for enzyme immobilization due to high mechanical stability, porous structure, large surface area, metal–semiconductor feature, and exceptional adsorption capability [75], [76]. In a report, PAMAM dendrimer coated magnetic multi-walled carbon nanotubes (m-MWCNTs) were used for oriented immobilization of Rhizomucor miehei lipase (RML). Results revealed that the m-MWCNTs-PAMAM matrix immobilized lipase showed recovery activity as high as 2808% with a 27-fold higher esterification activity than the free form. Under the optimal conditions, the biodiesel conversion by immobilized enzyme reached 94% from waste vegetable oil in a *tert*-butanol solvent system. Furthermore, m-MWCNTs-PAMAM bound lipase was easily recoverable without loss of any significant reduction in conversion efficiency even after 10 repeated conversion runs [77]. Magnetic graphene nanocomposites have also been the subject of growing attention in recent years [78]. A set of fascinating properties, including easy surface amendment, magnetic response, simple fabrication, high enzyme loading, and noticeable reusability have rendered them useful in many application such as catalysis, sensors development, lithium batteries, dye and ion removal, microwave absorption, supercapacitor electrodes, etc. [79], [80], [81]. For the first time, hyaluronic acid-coated MNPs-functionalized graphene oxide composites (GO-MNPs) for the immobilization of lipase B from *C. antarctica* (Fig. 4). With reference to the free biocatalyst, the storage stability of lipase-GO-MNPs was substantially improved. GO-MNPs immobilized lipase showed activity at elevated temperatures retaining over 90% of its recovered activity at 60 °C, whereas the soluble enzyme could preserve only 45% of its activity [82]. Rouhani et al. (2020) immobilized laccase from *T. versicolor* onto magnetic-graphene nanocomposites via glutaraldehyde crosslinking for the green preparation of sulfa drugs [83]. For this, magnetic GO nanocomposite was first fabricated by in situ co-precipitation method and then functionalized with APTES followed by cross-linking with glutaraldehyde. The performance of the immobilized nanobiocatalyst was markedly increased than that to free laccase in terms of stability and activity under the optimal conditions. It retained about 70% of its relative activity after incubating at 55 °C for 2 h, while only 48% of activity was recorded by the free laccase under identical time duration. Furthermore, the nanobioconjugate preserved higher than 85% of its activity after 20 days and possessed satisfactory recycling efficiency exhibiting 85% of its initial activity after eight recurrent runs.

## Small molecules and surfactants coating for enzyme immobilization

**Fig. 5:**
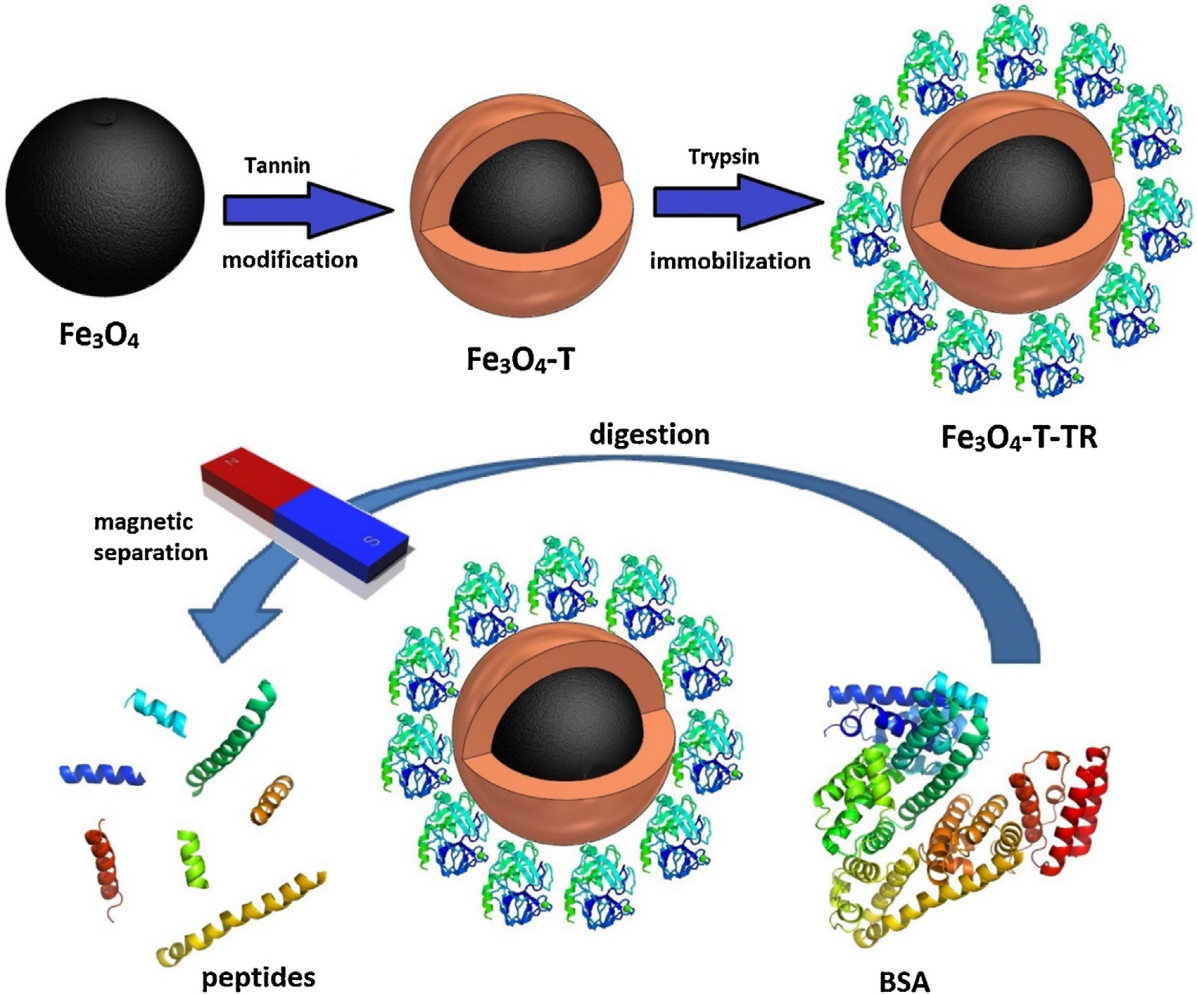
The illustration of modification and immobilization process on magnetic iron oxide nanoparticles for efficient BSA digestion. Reprinted from Ref. [94] with permission from Elsevier. License Number: 5092321139781.

**Fig. 6:**
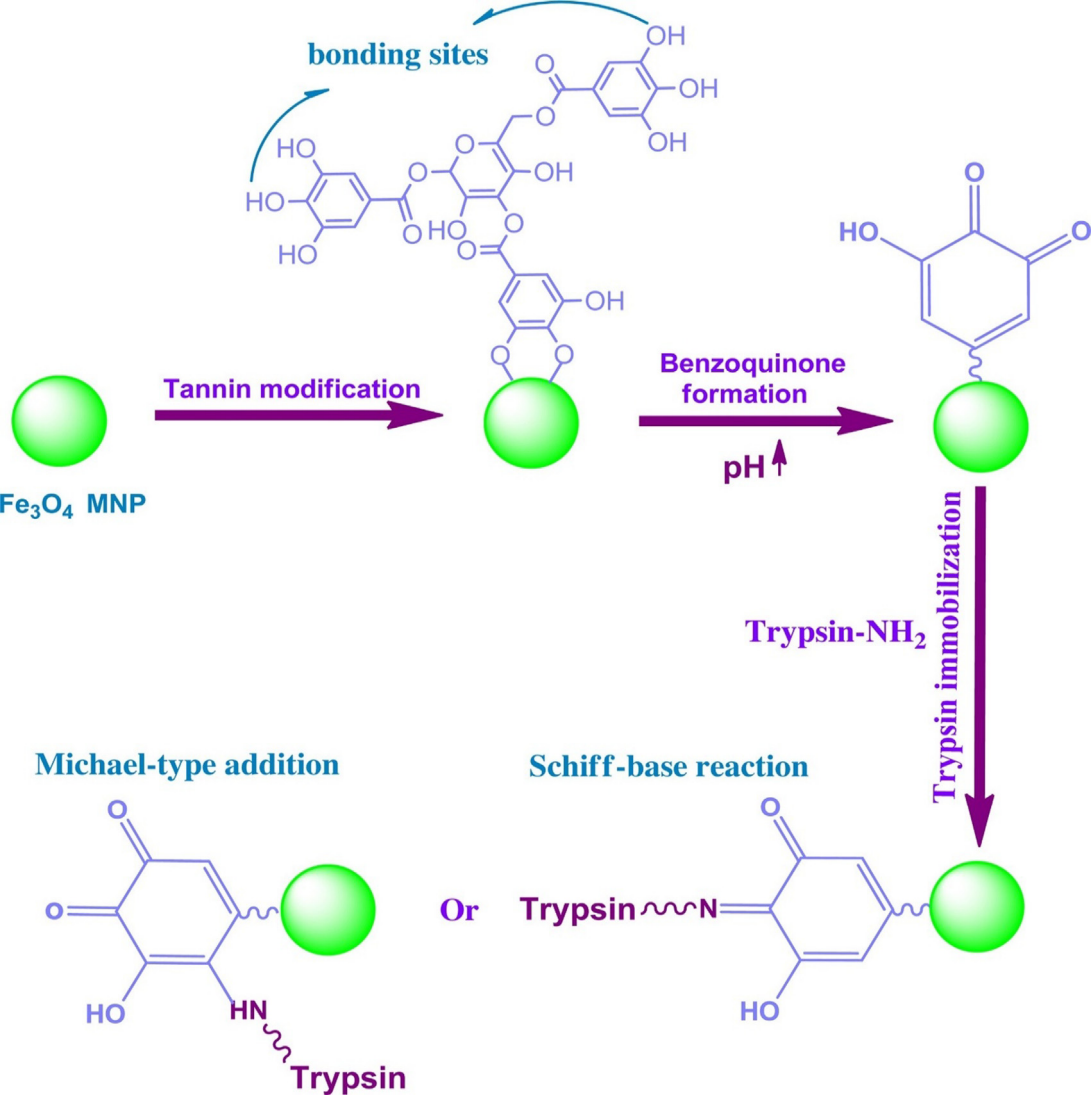
The chemical structure of pH-catalyzed oxidation of pyrogallol groups of tannin and subsequent binding reactions with the amines on trypsin. Reprinted from Ref. [94] with permission from Elsevier. License Number: 5092321139781.

A number of small organic molecules such as gallic acid, citric acid, amino acids, tannic acid (TA), cyclodextrin, vitamins, lauric acid, and dopamine are often employed for the modification of MNPs surface following various strategies to enhance the biocompatibility, and stability of these MNPs [84], [85], [86], [87]. One of the approaches is the direct inclusion of small organic molecules during fabrication. Tannic acid is a hydrophilic polyphenolic compound with wide applications in the production of resin, leather, and as polymer flocculants or coagulants for water remediation [88]. Tannic acid can be used in combination with Fe(III) for the modification of MNPs [89], [90]. During the use of tannic acid as a cross-linker, three galloyl groups from the tannic acid molecule react with each of Fe III ion forming an octahedral complex [91]. The tannin-metal complex formation is significantly influenced by the initial pH value. A tannin-metal mono-complex is generated at pH below 2, whereas a pH of 3–6 yields a bis-complex, and a stable tris-complex is formed at a pH of above 7. At ambient temperature, the intermingling of TA and Fe III with each other in water results in film formation. It is demonstrated that protein molecules with open and random coil-like conformations display a high affinity towards polyphenols compared with tightly folded structures [92]. Tannic acid-coated MNPs were applied as a support material to the immobilization of β-agarase, which exhibited greater pH and thermal resistance as well as appreciable recycling ability compared with the free counterpart. In addition, the immobilized β-agarase-TA-MNPs system was applied to prepare neoagaro-oligosaccharides with varying degrees of polymerization and antioxidant activities [93]. Atacan and coworkers adopted a solvothermal method to prepare MNPs and modified with tannic acid by a novel binding process for covalent immobilization of trypsin (Fig. 5) [94]. The trypsin was attached to tannin-modified MNPs by generating Michael-type addition or Schiff-base reaction among the quinone moieties present on the tannin NPS, which are produced by of pH-driven oxidation of pyrogallol groups of tannin, and the amino groups of the trypsin (Fig. 6). Thus, efficient immobilization was demonstrated by carrying out a novel process. Finally, the trypsin bound to tannin-coated MNPs was applied to successful enzymatic digestion of bovine serum albumin (BSA), where it showed a satisfactory digestion performance for BSA and egg white proteins.

As a natural molecule, gum arabic (GA) has demonstrated the ability to improve the colloidal stability of magnetic nanomaterials because of non-specific adsorption [95]. Coating with GA increases colloidal stability as well as incorporates reactive functionalities that aid in the coupling of biomolecules or enzymes. Mahmood et al. used GA as a coating agent to circumvent MNPs agglomeration and augment their biocompatibility making it an attractive carrier for CRL immobilization [96]. GA-coated MNPs (GA-MNPs) were fabricated by chemical co-precipitation technique and functionalized with glutaraldehyde for effective attachment of lipase enzyme onto this magnetic support. Before immobilization, the lipase surface was covered with different surfactants for stabilization of enzyme in its open form. The resulted surfactant-coated immobilized lipase bio-system was applied to produce ethyl isovalerate, a flavor ester. Among the different surfactants tested, non-ionic surfactants showed better contribution with the retention of 80% esterification yield in 48 h than that to corresponding anionic/cationic surfactants. Such improvement in activity might be ascribed to the interfacial activation of immobilized non-ionic surfactant-coated lipase conjugate. Moreover, surfactant-coated forms of the magnetic nanobiocatalyst preserved good catalytic activity after seven consecutive reuse cycles. In another report, the surface of MNPs was coated with gallic acid biomolecule. The phenolic moieties in gallic acid are attractive to nucleophiles, such as primary amines, making them capable of immobilizing enzyme molecules [97]. Furthermore, gallic acid presents the benefits of low cost, abundant availability, and biocompatibility. Trypsin (EC 3.4.21.4) was efficiently immobilized on the surface of easily fabricated gallic acid-coated MNPs, which offers a decent support matrix due to high accessible surface area and facile separation properties. As compared to the free enzyme, the immobilized form of trypsin presented high stability and retained high enzyme relative activity in alkaline pH conditions (pH range of 6 to 10.5) and a temperature range of 45 to 55 °C. It also showed appreciable storage stability retaining over 90% of activity after four months at 4 °C, while 60% of activity was recorded using the free enzyme under comparable conditions. After 8 continuous reuse times, the activity of the immobilized enzyme was found to 54.5% of its primary activity. It was capable of hydrolyzing BSA that manifests its application in the field of diagnostics, pharmaceuticals, food, and waste treatments [98].

## Metal-chelated MNPs for enzyme immobilization

**Fig. 7:**
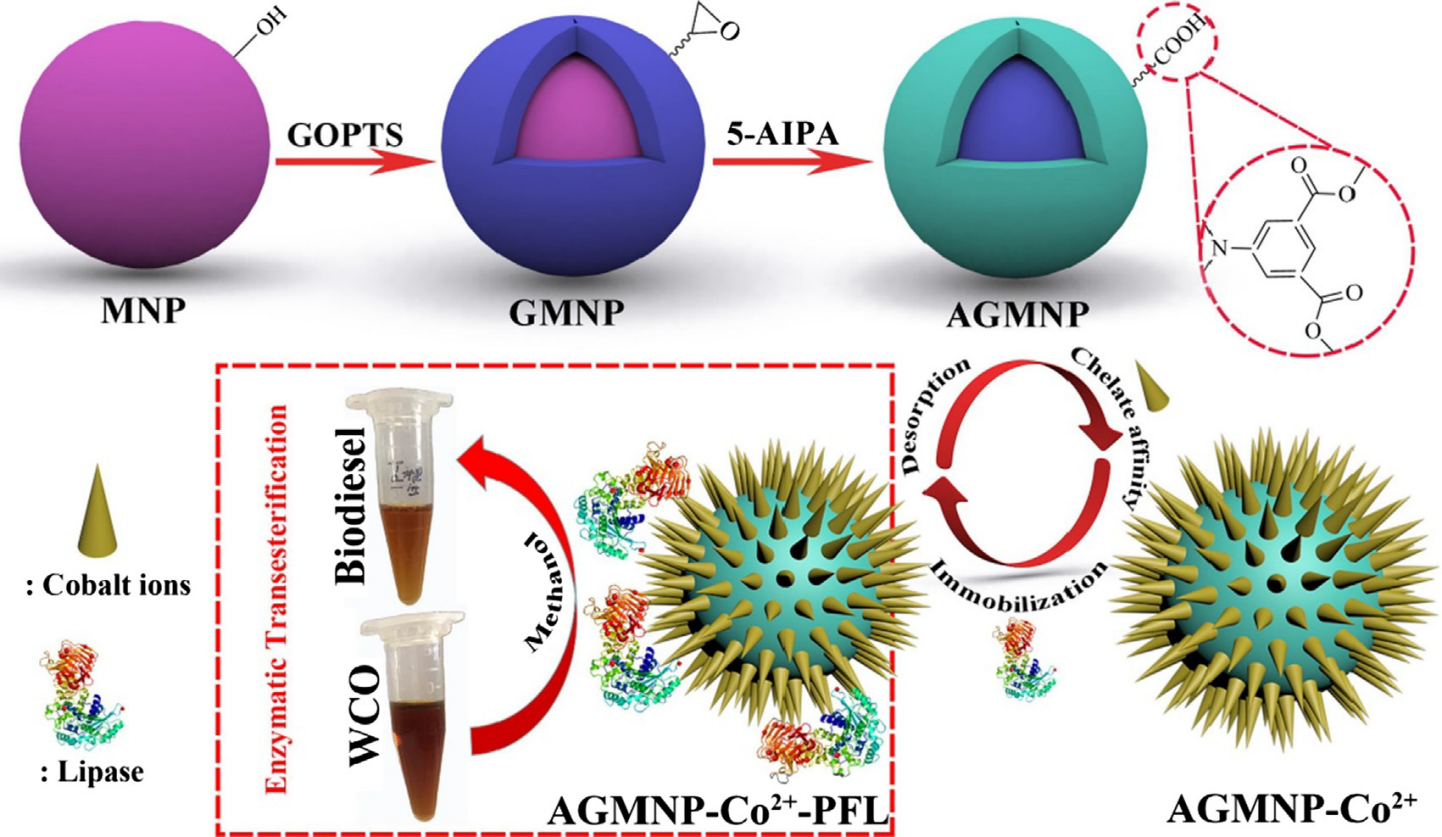
Schematic diagram of Co^2+^-chelated MNP preparation for use in reversible immobilization of lipase. Reprinted from Ref. [102] with permission from Elsevier. License Number: 5092321359009.

Surface functionalization of MNPs with metal elements may furnish an inert layer, which explicitly displays a core-satellite, core–shell, or dumbbell structural morphology. In addition, the functionalization of MNPs by metallic coatings results in improved compatibility and stability [99]. Loading of metal ions directly on the surface of support and chelation with enzyme molecules often display retention of high enzyme capacity and biocatalytic performance following the immobilization process [100]. Moreover, the metal-chelated affinity immobilization process is facile, fairly mild, and easy to perform, and the support could be recycled after desorption of the used enzyme. Hence, the metal-coated MNPs, integrating the benefits of paramagnetic property and immobilized metal ion affinity interaction, might have an incredible perspective for enzyme immobilization. Chen et al. (2014) synthesized agarose-coupled novel MNPs using by co-precipitation method under alkaline conditions [101]. MNPs were first coated with iminodiacetate using an epichlorohydrin agent followed by chelation with metal ions. The morphology and chemical properties of prepared support was analyzed by SEM, XRD, VSM, and FTIR. Among different metals ascertained, the Co^2+^-chelated agarose MNPs exhibited the highest β-glucosidase loading ability of 1.81 mg/g MNPs and attained the maximum recovered activity (117% per protein gram) for β-glucosidase immobilization. Immobilized bioconjugate displayed high operational and thermal stability and preserved over 90% of its preliminary activity after repeatedly using for 15 runs. A novel metal-chelating ligand, 5-aminoisophthalic acid (5-AIPA), was effectively coated onto MNPs, which were pre-decorated with (3-​Glycidoxypropyl) trimethoxy silane (GOPTS) for Co^2+^-chelated affinity immobilization of lipase from *P. fluorescens* [102]. Covalent coating of support surface with GOPTS was initially used to link MNPs beads containing hydroxy to create reactive epoxy groups for additional functionalization. Afterward, the condensation of GOPTS-coated MNPs with 5-AIPA caused the epoxy group to react with the amine group of aromatic carboxylic acid. Finally, Co^2+^-chelated AGMNPs were applied to immobilize lipase from Pseudomonas fluorescens, and adsorption–desorption of lipase enzyme achieved cyclic use of this support matrix (Fig. 7). Under the optimal environment, the resultant immobilized lipase possessed 95% conversion efficiency to synthesize biodiesel from waste cooking oil. It also retained higher than 80% of biodiesel yield after 10 repeated conversion cycles that demonstrate excellent operational performance. The designed support was readily regeneratable after the desorption of the inactivated enzyme and can be re-chelated with the Co(II) ions [102].

Wang et al. [87] prepared Ni^2+^-functionalized silica-coated MNPs (SiMNPs) using isocyanatopropyltriethoxysilane as a metal-chelating ligand to immobilize prolidase from *Escherichia coli* [103]. To this end, water-soluble nanocrystallites of iron oxide were first produced by the co-precipitation synthetic method, which was subsequently wrapped with a silica shell by reacting with tetraethoxysilane (TEOS) in aqueous ammonia. Further coupling of silica shell iron oxide nanoparticles with the chelating agent 3-(triethoxysilyl)propyl isocyanate-nitrilotriacetic acid (ICPTES-NTA) and NiCl_2_ results in the formation of fully-armed Ni^2+^-functionalized silica-wrapped MNPs (NiNTASiMNPs). The resultant synthesized NiNTASiMNPs were employed as an affinity probe to adsorb His6-*Ec*PepQ. Characterization of native and NiNTASiMNPs-bound enzyme revealed that His_6_-EcPepQ@NiNTASiMNPs enzyme showed greatly improved activity at elevated temperature of 70 °C and a wider pH range of 5.5 to 10 than that to free counter form. It also displayed enhanced stability when stored for 2 months and reusable for over 20 cycles by retaining 80% of its original activity. Moreover, the immobilized magnetic nanobiocatalyst was applied to degrade organophosphorus compounds, including diethyl *p*-nitrophenyl phosphate (ethyl paraoxon) and dimethyl p-nitrophenyl phosphate (methyl paraoxon). Results demonstrated dimethyl p-nitrophenyl phosphate as a preferred substrate for degradation by immobilized prolidase.

## Mesoporous material-modified MNPs for enzyme immobilization

Mesoporous materials have been recognized as prodigious carriers for enzyme immobilization due to a set of desired features including designable pore size, huge surface area, non-toxicity, greater pore volume, and thermo-chemical strength [104]. Enzymes can be attached on the surface or trapped within the pores of mesoporous matrices by covalent coupling, cross-linking, or simple physical adsorption. A large number of various organosilanes are utilized to insert cyano, amino, epoxy, or sulfhydryl functionalities on the surface of mesoporous materials for efficient enzyme attachment [105]. The introduction of these functional groups generates many reactive sites to bind biomolecules and thus enhance the loading capacities. Furthermore, the penetration of organic groups into the mesoporous channel reduces the pore volume or size, resulting in the prevention of enzyme leaching. Likewise, Muñoz-Pina and coworkers (2018) also examined the polyphenol oxidase (PPO) immobilizing ability of many thiol-modified mesoporous silica materials with diverse geometrical shapes, structures, and pore sizes. UVM-7 is a silica-based mesoporous material that comprises of both mesopores and textural pores. It also has shown potential for enzyme immobilization such as polyphenol oxidase in both real and model systems [106]. More recently, the same group fabricated five different nanostructured materials to inspect their capability to increase the activity of the PPO enzyme. All these materials were based on a mesoporous silica material (UVM-7 support) and introduced with different functionalities (i.e. amine, alkane, isocyanate, pyridine, and carboxylic acid) to evaluate PPO immobilization capacity. Except for the carboxylic acid-modified nanomaterial, all other functionalized nanosupports offered high enzyme loading abilities and the immobilization rate increases with functionalization. Interestingly, amine-carrying support material captured not only the PPO enzyme but also sequestrated the resultant oxidation products. This nanomaterial was corroborated by reacting with fresh apple juice in which no browning occur even after exposure to 1.5 h in the presence of oxygen [106]. Furthermore, the modification of MNPs with mesoporous materials including silica helps in efficient encapsulation/adsorption of enzymes, due to various reasons including the presence of hollow spheres with mesoporous walls, ability of forming unique core/shell system etc [107]. For instance, khorshidi et al., reported the successfull immobilization of cross-linked cellulase aggregates (CLEA) on the amine-functionalized Fe_3_O_4_@silica core–shell magnetic nanoparticles (MNPs) [108]. In another study, Wang et al., have exploited the mesoporous properties of silica nanoparticles to fabricate wormhole framework structured mesoporous silica-magnetite nanocomposites [109]. The magnetic nanocomposites were prepared by using tetraethyl orthosilicate as the silica source and amine terminated Jeffamine surfactants as template. The nanocomposites were modified by chelating with copper to further enhance the adsorption capacity. The Cu^2+^ chelated magnetic nanocomposite showed higher adsorption capacity of 98.1 mg g^−1^ -particles and activity recovery of 92.5% for laccase via metal affinity adsorption. More recently, core − shell structured magnetic mesoporous silica microspheres with ultra-large mesopores were developed using a controllable solvent evaporation induced solution-phase interface co-assembly approach [110]. For this purpose. large-molecular-weight amphiphilic block copolymers poly(ethylene oxide)-blockpoly(methyl methacrylate) (PEO-b-PMMA) and small surfactant cetyltrimethylammonium bromide were employed as co-templates, which co-assembled with silica source in tetrahydrofuran/water solutions. The as-prepared composite was used to immobilize trypsin, which demonstrated a high loading capacity of 80 μg/mg. In another study, mesoporous yolk–shell nanoparticles with a movable Fe_3_O_4_ core inside the hollow capsules, with two different morphologies were fabricated using a template-assistant selectively etching method [111]. The composites were applied as carriers for Candida rugosa lipase (CRL) immobilization.

## MOF-modified MNPs for enzyme immobilization

Amongst nanostructured materials, MOFs are considered an inspiring group of support materials for enzymes encapsulation owing to their exceptionally larger surface area, pore size uniformity, tunable surface and porosity, easy recovery, functional and structural versatility, and high thermal and chemical stability [112], [113]. Surface adsorption, co-precipitation, and pore encapsulation are three notable immobilization strategies that can be used to embed enzymes onto MOFs. Co-precipitation strategy can protect enzyme molecules from thermal, biological, and chemical degradation by embedding enzyme molecules within MOFs, and resultantly, generate immobilized enzyme with improved stability [114]. The use of MNPs-MOF nanocomposites produced by integrating features of both Fe_3_O_4_ NPs and MOFs have emerged as novel platforms for immobilizing a diverse array of enzymes [115], [116]. Generally, MNPs-MOF nanocomposites are produced by the reaction between metal ions and organic ligands using carboxylated Fe_3_O_4_ NPs. Wang et al. (2016) introduced an efficient, facile, and environmentally-responsive approach for the preparation of magnetic MOF nanocomposite [117], Fe_3_O_4_@MIL-100(Fe). For this, carboxyl-modified Fe_3_O_4_ nanorods were incorporated with three-dimensional MIL-100(Fe) nanocrystals. The as-prepared composite microsphere showed the substantial surface area and strong magnetic properties, which make them attractive contenders for enzyme immobilization. The CRL was immobilized onto Fe_3_O_4_@MOF core–shell microspheres by covalent attachment (strategy I) and metal-ion affinity interfaces (strategy II). The immobilized nanobiocatalytic system reserved 65% of its preliminary activity at 65 °C for the hydrolysis of olive oil over 6 h. It retained over 60% of remaining activity after 10 reiterated catalytic runs and presented a significant improvement in biocatalytic activities at broader temperature and pH ranges than that to the free enzyme. High enzyme loading capacity was ascribed to large pore size and surface area combined with the occurrence of free carboxyl groups and unsaturated metal sites in MOFs.

A novel magnetically active Ni-based MOF composite was prepared for efficient separation and immobilization of enzymes [118]. For this, a facile one-pot hydrothermal method was adopted to develop Ni-based MOF nanorods (Fe_3_O_4_/Ni-BTC) with a good magnetic response by the entrapment of citric acid-coated MNPs on Ni-BTC. Characterization revealed that these nanocomposites were fabricated in the form of nanorods, which contained MNPs on their surface. A variety of different interactions played a key role in enzyme immobilization, such as hydrogen bonding, electrostatic attraction, hydrophobic forces, and affinity between histidine tags and Ni^2+^. Based on the mechanistic understanding of these nanocomposites, a new approach was proposed for the immobilization of S-adenosylmethionine synthetase (SAMS), which showed high stability against extreme pHs and elevated temperature, and showed noteworthy repeatability profile after immobilization process [118].

Laccase from white-rot fungi was immobilized, for the first time, onto amino-functionalized magnetic MOF, Fe_3_O_4_-NH_2_@MIL-101(Cr) [119]. Immobilized laccase prepared by the covalent bonding and adsorption method revealed high immobilization yield, largely recovered activity, and better endurance to low pH and elevated temperature regimes. It also showed excellent storage stability holding over 85% of its initial bioactivity after storage of 28 days. At an extreme temperature of 85 °C, Fe_3_O_4_-NH_2_@MIL-101(Cr) bound biocatalyst presented about 50% of the remaining activity even after heating for 6 h. The tolerance of immobilized laccase was greatly improved in organic solvents like methanol. Finally, Fe_3_O_4_-NH_2_@MIL-101(Cr)-based biocatalytic system led to the rapid removal of 2,4-dichlorophenol, reaching the removal efficiency to 87%. After the reaction, laccase can be easily recovered by the mean of a magnet from the complex reaction solution. All these features make MOF-based MNPs nanocomposite a novel nanoplatform for immobilizing an array of enzymes with high catalytic activity and great biotechnological potential [119]. Table 2 portrays a current summary of the use of surface-engineered magnetic nanoparticles as novel paradigms for enzyme immobilization.

**Table 2:** Some examples of surface-coated magnetic nanoparticles as support materials for enzyme immobilization and their applications.

Magnetic nanocarrier	Enzyme	Functional reagent	Improved properties	Application	References
MNPs	*Pseudomonas fluorescens* lipase	Co^2+^	Immobilized lipase possessed 95% conversion efficiency to synthesize biodiesel from waste cooking oil. Excellent operational performance retained higher than 80% of biodiesel yield after 10 repeated conversion cycles.	Biodiesel production	[102]
Fe_3_O_4_-NH_2_@MIL-101(Cr)	Laccase from white rot fungi	MIL-101	High recovered activity, and better endurance to low pH and elevated temperature regimes. Excellent storage stability retaining over 85% of its original bioactivity after storage of 28 days. At an extreme temperature of 85 °C, Fe_3_O_4_-NH_2_@MIL-101(Cr) bound biocatalyst presented about 50% of the remaining activity even after heating for 6 h. Rapid removal of 2,4-dichlorophenol, reaching the removal efficiency to 87%.	Removal of phenolic compounds	[119]
Agarose-coupled novel MNPs	*β*-glucosidase from sweet almond	Co^2+^	Immobilized bioconjugate displayed high operational and thermal stability, and preserved over 90% of its preliminary activity after repeatedly using for 15 runs.	Production of aromatic compounds Ethanol from cellulosic agricultural residues	[101]
MNPs-functionalized graphene oxide composites	Lipase B from *Candida antarctica*	Hyaluronic acid	As compared to the free enzyme, the storage stability of lipase-GO-MNPs was substantially improved. GO-MNPs immobilized lipase showed activity at elevated temperatures retaining over 90% of its recovered activity at 60 °C, whereas the free enzyme retained only 45% of its activity under the same temperature conditions.	Biodiesel production, pharmaceuticals and cosmetic industry	[82]
MNPs	Porcine pancreatic lipase and penicillin G acylase	Cellulose	Improved catalytic activity and stability of immobilized enzymes. Easy separation of immobilized enzymes from the reaction system.	Enzyme immobilization	[120]
MNPs	*β*-agarase	Tannic acid	Immobilized β-agarase, exhibited greater pH and thermal resistance as well as appreciable recycling ability compared with the free counterpart. The immobilized β-agarase-TA-MNPs system was applied to prepare neoagaro-oligosaccharides with varying degrees of polymerization and antioxidant activities	Preparation of bioactive neoagaro-oligosaccharide	[93]
Trichlorotriazine-functionalized MNPs	Pectinase	Polyethylene glycol	Immobilized enzyme presented improved satisfactory operational stability, improved catalytic efficiency, and easily recyclability in multiple cycles. Augmented pH and thermal stability profile than the free enzyme. Retained up to 94% and 55% of its actual activity after storage for 125 days at 25 °C, and 10 repeated catalytic runs, respectively. A prominent reduction in turbidity of pineapple juice (up to 59%) after treatment with the immobilized enzyme.	Fruit juice clarification	[65]
Fe_3_O_4_@MIL-100(Fe)	*Candida rugosa* lipase	MIL-100(Fe)	Immobilized nanobiocatalytic system retained more than 65% of its original activity at 65 °C for the hydrolysis of olive oil in 6 h. It retained over 60% of residual activity still after 10 repeated catalytic runs. Presented a significant improvement in biocatalytic activities at broader temperature and pH ranges than that to the free enzyme.	Transesterification and synthesis of esters	[117]
MNPs	Cholesterol oxidase	Silica	In contrast to the soluble enzyme, the covalent immobilization of biocatalyst was able to retain about 50% of its activity.	Development of biosensing components	[121]
MNPs	Glucose oxidase	Silica	Immobilized bioconjugate preparation maintained over 95% and 90% of its original activity after storage for 45 days, and 12 consecutive reaction cycles. Substantial improvements in thermal stability profiles were also recorded at high temperatures up to 80 °C. Moreover, the immobilized biocatalyst was less likely to be affected by alterations in pH values	Biomedical applications	[33]
MNPs	Phospholipase D	Silica	Increased tolerance of immobilized enzyme to high temperature. Catalytic activity of the immobilized biocatalyst retained to be 40% after eight recycles.	Synthesis of functional phosphatidylserine	[122]
MNPs film	Horseradish peroxidase from horseradish cv. Balady	Polymethyl methacrylate	Excellent reusability retaining 78.5% of its initial activity after 10 repeated cycles. High stability of the immobilized HRP against metal ions, a high urea concentration, isopropanol, and Triton X-100. Efficient removal of phenol in the presence of hydrogen peroxide.	Removal of wastewater aromatic pollutants	[123]
Fe_3_O_4_–graphene nanocomposite	*Trametes Versicolor* laccase	APTES	Stability and activity of the immobilized nanobiocatalyst was markedly increased than that to free laccase. Retained about 70% of its relative activity after incubating at 55 °C for 2 h, while only 48% of activity was recorded by the free laccase under identical time duration. Nanobioconjugate preserved higher than 85% of its activity after 20 days of storage and possessed satisfactory recycling efficiency exhibiting 85% of its original activity after eight repeated cycles.	Green preparation of sulfa drugs	[83]
Biomimetic silica-MNPs hybrid nanocomposite	*β*-glucuronidase from *Patella vulgata* limpets	silica	Superior storage, thermal, and operational stability of the enzyme immobilized in the composite material. Different bioconjugates with MNPs and Si maintained 40% of their original activities at a high temperature of 80 °C after 6 h, while the free form of enzyme dropped over 90% of its activity within 10 min.	Pharmaceutical and food industry	[34]
Fe_3_O_4_/Ni-BTC	S-adenosylmethionine synthetase from *Thermus thermophilus* HB27	Citric acid	Iimmobilized enzyme was more stable against temperature variation (by nearly 8-fold in an 80 °C water bath after 2 h) and extreme pH (by nearly 1.3-fold at pH 3). Excellent reusability after immobilization with high efficiency and stability.	Biosynthesis of S-adenosylmethionine	[118]
Amino-functionalized MNPs	Alkaline protease from *Bacillus licheniformis*	APTES	Excellent operational stability retaining 50.1% of its initial activity after 10 cycles. Efficient catalytic hydrolysis of oat bran into oat polypeptides.	Preparation of oat polypeptides	[124]
Ni^2+^-functionalized MNPs	Prolidase from *Escherichia coli*	Silica	Improved activity at elevated temperature of 70 °C and a wider pH range of 5.5 to 10 than that to free counter form. Enhanced stability at storage for 2 months and reusable for over 20 cycles by retaining 80% of its original activity. Degradation efficiency for organophosphorus compounds.	Hydrolysis of organophosphorus compounds	[103]
MNPs	*Candida rugosa* lipase	Alkyl silane	Increased catalytic activities of lipases after immobilization. Good stability and recycling ability retained 65% of its initial activity after seven repeated cycles.	Enzyme immobilization	[125]
NPs	Horseradish peroxidase from horseradish cv. Balady	Carbon	Enzyme-based novel amperometric electrode	H_2_O_2_ sensing	[73]
MNPs	Lipase from *Thermomyces lanuginosus*	Polydopamine	A broader pH and temperature adaptability as compared to the free enzyme. Improved pH, thermal, and solvent tolerance stabilities compared to the free enzyme.	Biodiesel production, organic synthesis, and environmental protection	[126]
MNPs	Cellulase from *Aspergillus fumigatus*	–	Immobilized enzyme retained 56.87% of its maximal activity after 6 h of incubation at 60 °C. Efficient hydrolysis of pre-treated rice straw with saccharification efficiency of 52.67%. Reutilization for up to four saccharification cycles with retention of 50.34% activity.	Enzymatic saccharification of rice straw	[127]
Magnetic carbon nanotubes	Glucoamylase from *Aspergillus niger*	Poly(amidoamine)	superior stability and reusability, without compromising the substrate specificity of free glucoamylase	Starch processing and glucose production	[128]
Metallic nanomagnets	α-chymotrypsin, lipase B, and *β*-glucosidase	Carbon	Immobilized bioconjugate preparations showed good stability and catalytic performance and could be recyclable from milliliter to liter volumes in short recycling durations.	Analytical immunoprecipitation and cell separation	[72]
MNPs with long alkyl chains	*Candida rugosa* lipase	poly- N,N diethylaminoethyl-acrylamide	Nanoimmobilized biocatalytic system with the longest alkyl chains presented superior tolerance to high temperature (ranging from 25 to 70 °C) than that to the free form of lipase. It also showed good recyclability in four successive cycles and conveniently recovered by a simple magnetic separation.	Biodiesel production, food processing , cosmetic and pharmaceutical industry	[49]
Divinyl sulfone superparamagnetic nanoparticles	Lipase from *Thermomyces lanuginosus*	Polyethyleneimine	Good enantioselectivities with high catalytic activities in the reaction medium at pH 7.0. Excellent operational stability in the esterification reaction obtaining up to 61 % conversion after the seventh reaction cycle.	Biodiesel production, food processing , cosmetic and pharmaceutical industry	[129]
Superparamagnetic nanoparticles (Fe_3_O_4_)	Lipase from *Thermomyces lanuginosus*	Polyethylenimine, APTES, and Glutaraldehyde	The SPMN (superparamagnetic nanoparticle) @APTES covalent preparation had around 450 min of half-life time at pH 7.0 and 70 °C while that of the free enzyme was 46 min. The conversion attained was 50% and the enantiomeric excess of the product was 99%.	Recovery of the biocatalyst	[130]
MNPs	Alcohol dehydrogenase	Carboxymethyl dextran	In contrast to the free form of ADH that dropped 70% of its original activity at 20 °C, and complete loss of its activity at 40 °C after 24 h. Nanoimmobilized biocatalyst retained more than 50%, and 75% of its remaining activity at 20 °C and 40 °C, respectively, under the same incubation period of 24 h.	Chemical industries	[48]
Fe_3_O_4_/SiO_2_/NH_2_	L-asparaginase	APTES, and Glutaraldehyde	ASNases were more stable in a wide range of pH and temperature values under the optimum reaction conditions. High stability at an elevated temperature of 50 °C for 3 h. Free form of enzyme showed only 30% of its original activity after preserving at 4 °C for 1 month, whereas Fe_3_O_4_/SiO_2_/NH_2_ ASNase preserved above 78.9% of its preliminary activities. Outstanding functioning stability after 17 consecutive batch cycles.	Anti-leukemia chemotherapy	[31]
Fe_3_O_4_/SiO_2_/COOH	L-asparaginase	APTES, and Glutaraldehyde	High stability in a wide range of pH and temperature values. Preservation of 56.5% of its initial activity. Outstanding operational stability in several consecutive cycles.	Anti-leukemia chemotherapy	[31]
Magnetic graphene nanocomposite	*Trichoderma reesei* cellulase	Chitosan	With regard to the soluble enzyme, the nanobiocatalytic system showed highly enhanced bioactivity and retained over 75% of its actual activity. After the immobilization process, a substantial widening in pH, storage, and thermal stability were obtained. The immobilized cellulolytic enzyme was capable of maintaining a high degree of its original activity after repeatedly using for 8 cycles.	Saccharification of microcrystalline cellulose	[60]
Sebacoyl-modified MNPs	Lipase B from *Candida antarctica*	Chitosan	High activity up to 10 repeated catalytic cycles under the optimized conditions (n-hexane, vinyl acetate, 45 °C).	Enzymatic Kinetic Resolution of Racemic Heteroarylethanols	[61]
MNPs	*β*-glucosidase from *Thermotoga maritima*	Chitin, chitosan, and sodium alginate	Marked reusability of the nanobiocatalytic system in several successive batches for GOS synthesis without a substantial loss of enzyme activity. Immobilized enzyme showed operational stability under varying pH, temperature, storage, and thermal conditions.	Galacto-oligosaccharide production	[63]
Iron oxide magnetic nanocomposite	Manganese peroxidase from *Anthracophyllum discolor*	Chitosan	The nanobioconjugate preparation retained its activity and demonstrated recycling ability in 5 consecutive reaction cycles.	Decolorization of textile wastewater	[62]
Fe_3_O_4_@SiO_2__EDTA-TMS	Laccase	EDTA-Cu (II)	Good operational stability of the immobilized enzyme presenting 73% of its initial activity after five sequential reactive cycles. Successfully applied to the degradation of Indigo Carmine dye	Biocatalysis and biosensors	[131]
MNPs	Tyrosine	Tannic acid	Enzymatic digestion of bovine serum albumin	Protein digestion	[94]
MNPs	Tyrosine	Gallic acid	Immobilized trypsin presented high stability and retained high enzyme relative activity in alkaline pH conditions (pH range of 6 to 10.5) and a temperature range of 45 to 55 °C. It also showed appreciable storage stability retaining over 90% of its original activity after storage for 4 months at 4 °C. After 8 continuous reuse times, the activity of the immobilized enzyme was found to 54.5% of its primary activity.	Diagnostics, pharmaceuticals, food, and waste treatments	[98]
MNPs	*Candida rugosa* lipase	Gallic acid	Improved esterification activity. Surfactant-coated forms of the magnetic nanobiocatalyst preserved good catalytic activity after seven consecutive reuse cycles.	Production of multicycle ethyl isovalerate	[96]
Fe_3_O_4_@silica yolk-shell nanospheres	Catalase from bovine liver	TMOS, APTES	Enhanced recycling efficiency and high resistance to heat, proteolytic agent, and denaturants.	Enzyme shielding	[132]
Fe^3+^-TA@ Fe_3_O_4_/SiO_2_-catalase	Catalase from bovine liver	TMOS, APTES	Improved stability and efficient recycling ability	Shielding effect to protect enzymes from thermal, biological, and chemical degradation	[133]
Fe_3_O_4_@mSiO_2_	Nitrile hydratase	Glutaraldehyde	Improved pH, thermal, mechanical and storage stability	Catalysis production of nicotinamide	[134]
CA-Fe_3_O_4_ NPs	Lipase	Citric acid	Excellent long-term storage stability and increased activity at high temperature and pH	Enzyme immobilization	[4]

MNPs—Magnetic nanoparticles; TMOS— Tetramethyl orthosilicate.

## Biomedical applications of surface coated MNPs.

### Detection, diagnostics and therapy

MNPs have often been widely used in several biomedical applications such as, biosensing, medical diagnostics, drug delivery, gene transfer etc. [135]. Among various extraordinary physicochemical properties of MNPs including fluorescence, photoacoustic effects, hyperthermia, magnetism, and photothermal properties [136], [137]. Particularly, enzyme-like catalytic properties of MNPs, such as peroxidase-like activity of iron oxide NPs, have received immense interest in the field of biomedicine [138]. Due to these enzymatic properties, in some cases modified MNPs are referred as nanoenzymes (enzyme mimetic MNPs), which are artificial enzymes with highly effective enzyme-like properties [139], [140]. Recently, these materials have gained considerable interests as they are easy to prepare, controllable in size and adjustable in function [141]. Besides, they offer stability and multifunctionality when compare to conventional enzymes and thus offer versatile applications in the field of biomedicine [138]. Enzyme mimetic MNPs demonstrate high stability even under harsh conditions including high temperatures, high acidic and basic environments. For example, Fe_3_O_4_ NPs stabilized with peroxidase-like casein showed excellent stability in wide range of pH between 1 and 12, and temperatures from 4 to 90 °C [142]. The as-prepared casein-MNPs were applied to catalyze the oxidation of a peroxidase substrate 3,3′,5,5′-tetramethylbenzidine (TMB) by H_2_O_2_ to the oxidized colored product which provides a colorimetric detection of H_2_O_2_. Contrarily, HRP enzyme did not exhibit any activity after treatment at lower pH (less than 5) and rapidly lost its activity as the temperature increased to 40 °C. Due to the smaller size of NPs and high surface area enzyme mimetic MNPs also offer excellent biocatalytic activity which can be further tuned by controlling the size and morphology of nanoparticles. This phenomenon has been successfully demonstrated by Gao et al., who has developed a novel immunoassay involving antibody-modified magnetite nanoparticles for the successful capturing, and separation of wastes in the treatment of wastewater. The small sized enzyme mimetic MNPs have exhibited highest catalytic activity in the order 30 nm greater than 150 nm greater than 300 nm [143]. In addition, the enzymatic activity of enzyme mimetic MNPs also dependent on the shape of NPs. Different shapes of iron oxide nanostructures like octahedral, spheres, and triangular plates have showed different peroxidase-like activities [144]. Apart from enzyme-like activity, iron oxide based enzyme mimetic MNPs also possess excellent superparamagnetism properties which can be exploited for multipurpose performances. For examples, the enzyme like activities of MNPs can be utilized to replicate peroxidase and catalase activities at acidic and neutral pH, respectively. Chen et al., have demonstrated the process of controlling the ability of free radicals under intracellular microenvironment through pH-dependent peroxidase-like and catalase-like activities of iron oxide NPs [145]. They have investigated the interaction of nanoenzyme with H_2_O_2_ within cells, the results revealed a concentration-dependent cytotoxicity on human glioma U251 cells, and they could dramatically enhance H_2_O_2_-induced cell damage. This pH controlled enzyme like activity can be effectively utilized under special circumstances like tumors or biofilms. Similarly, the enzyme mimetic MNPs offers great opportunity in other biomedical applications. Mostly, the peroxidase like activity of these materials have been utilized to enhance the signal detection during colorimetric reactions and generate free radicals to kill bacteria and cells or interfere ROS level. One of the primary applications of enzyme mimetic MNPs is the replacement of horseradish peroxidase (HRP) in enzyme-linked immunosorbent assay (ELISA) and other HRP-related molecular detection. Such as the development of MNPs based immunosorbent assay by Gao et al., using chitosan-modified magnetite nanoparticles to replace enzymes in conventional ELISA configurations [35].

Besides, these enzyme mimetic MNPs based novel immunoassays have also been used to detect various antigens or pathogens including human chorionic gonadotropin (HCG), IgG and epidermal growth factor receptor (EGFR) [138]. Apart from detections, enzyme mimetic MNPs have also been used for tumor diagnosis and therapy. Such as, magneto-ferritin nanoparticles (M−HFn) which targeted and visualized affected tissues (tumor) without using any targeting ligands or contrast agents [146]. In addition, these materials have also been used for the direct elimination of tumors. Particularly, iron oxide NPs have been very useful in this regard, they facilitate the generation of toxic radicals by catalyzing which affect tumor viability. However, in many cases the amount of H_2_O_2_ is not sufficient to initiate toxicity [147]. To enhance this, H_2_O_2_ is directly injected into the body or combine with an enzyme to generate H_2_O_2_ using in vivo substance as substrate. Indeed, the later process is more suitable compare to the direct injection of H_2_O_2_, which may cause unwanted damage to local tissues.

### Bioelectronics

In this field, advance functional bio-devices such as biosensors, biofuel cells etc., are created by integrating biomolecules including enzymes with electronic systems. Recent advancements in the field of nanobiotechnology have enabled to create highly sensitive biomedical devices with advance functionalities. Biofuel cells involve biological materials, including proteins, microorganisms, enzymes etc., as catalysts to convert chemical energy into electrical power. The enzyme based biofuel cells typically consist of isolated enzymes as catalysts, anode, cathode and electrolyte materials [148]. So far, various oxidoreductase enzymes such as glucose dehydrogenase, aldehyde dehydrogenase, glucose oxidase etc., have been used in different types of enzymatic biofuel cells which have been applied as a power source for portable or implantable electronics, including pacemaker etc. For example, a ternary conducting nanocomposite, involving Fe_3_O_4_ MNPs, carbon nanotubes (CNT), gold nanoparticles (Au) and a conducting polymer polypyrrole (PPy), was developed and applied as the electrode support for the immobilization of glucose oxidase (GOD) [149]. The resulting composite improved the bioelectrocatalysis of the enzyme towards oxidation of glucose. In another study, magnetic carbon-encapsulated iron nanoparticles (CEINs) immobilized with laccase (Lc) and 1,4-naphthoquinone (NQ) and fructose dehydrogenase (FDH) were used for the fabrication of bioelectrodes in a biobattery and a biofuel cell [150]. In the device, the glassy carbon bioanode was coated with carbon-encapsulated iron nanoparticles, 1,4-naphthoquinone, fructose dehydrogenase, and Nafion, while the cathode was modified with carbon-encapsulated magnetic nanoparticles and laccase in the Nafion layer. A maximum power of 78 µW/cm^2^ at the voltage of 0.33 V and under 20 kΩ resistance, and the open-circuit voltage was 0.49 V was used. These enzymes worked effectively in the biofuel cell, and laccase also effectively worked in the biobattery.

Another application of enzyme coated MNPs are the fabrication of biosensors involving sensitive biological entities like antibodies, cell receptors, enzymes, transducers and, detectors associated with signal processing and electronics [32]. Commonly used MNPs and enzymes-based biosensors are glucose sensor for blood-sugar tests. Particularly, the process of enzyme immobilization is crucial to improve the sensitivity of the biosensors, which has been considerably progressed due to the advancement in the field of nanobiocatalysis [151]. Recently, Pakapongpan et al., have successfully immobilized glucose oxidase (GOD) on reduced graphene oxide (RGO), which is covalently conjugated to magnetic nanoparticles (Fe_3_O_4_ NPs) to obtain highly selective and stable glucose biosensor [152]. The proposed biosensor showed fast amperometric response (3 s) to glucose with a wide linear range from 0.05 to 1 mM, a low detection limit of 0.1 μM at a signal to noise ratio of 3 (S/N = 3) and good sensitivity (5.9 μA/mM). Similarly, a biosensor based on nanomagnet-silica core–shell conjugated to organophosphorous hydrolase (OPH) enzyme was designed for detection of paraoxon [153]. In another study, a novel core–shell Fe_3_O_4_@poly(dopamine) MNPs hybrid was fabricated using an in situ self-polymerization method [38]. The as-prepared nanohybrid was used as a solid support for the covalent immobilization of horseradish peroxidase (HRP), and the resulting biofunctionalized MNPS were employed to fabricate an amperometric biosensor for H_2_O_2_. The enzyme biosensor showed a high sensitivity of 442.14 mA M^−1^ cm^−2^, a low limit of detection of 182 nM, a wide linear range from 6.0 × 10^−7^ to 8.0 × 10^−4^ M and high stability for 1 month.

### Proteomics

This field involve proteins analyses which maybe play a significant role in the field of biomarkers, drug treatment, medical diagnostics etc. Recently, this field has progressed due to the advancement of technologies in mass spectrometry analysis, protein quantification and bioinformatics data analysis. Particularly, Mass spectrometry plays a crucial role in large-scale protein analysis, but the downstream proteome analysis is typically effected by the cumbersome sample preparation methods [154]. This is typically simplified by the process of protein digestion in-solution using proteolytic enzymes such as trypsin. Trypsin (Try) is typically immobilized on a solid support including MNPs to make it more friendly for the mass spectrometric analysis [155], [156]. A method of combining trypsin-immobilized MNPs and microwave-assisted protein digestion is reported to study the proteins of human lens tissue, which were identified by liquid chromatography and mass spectrometry [157]. But, unprotected MNPs are very unstable and can be readily oxidize in atmospheric conditions [158]. Therefore, MNPs are often functionalized to protect them against oxidation and to facilitate enzyme immobilization using various stabilizing agents including polymers or other compounds containing amino (–NH_2_), hydroxide (–OH), carboxylic acid (–COOH) and phosphate groups [159], [160]. For example, polyaniline-coated nano-Fe_3_O_4_/carbon nanotube composite exhibited enhanced digestion efficiency of Try due to the high surface area-to-volume ratio of nanoparticles, which increased interaction between Try and the substrates (proteins) [161]. In another study, MNPs were stabilized with polyvinyl alcohol and activated with glutaraldehyde for trypsin immobilization [162]. Pristine (without MNPs) and immobilized trypsin on MNPs showed optimum activity at pH 6.0, 30 °C and pH 7.0, 40 °C, respectively, while trypsin immobilized MNPs was more stable than the free enzyme at 40 °C. Indeed, a recent study has demonstrated that trypsin immobilized on MNPs at the nano-scale performs better than the commercially available macroparticles counterpart [154].

A quantitative proteomics method based on liquid chromatography coupled to mass spectrometry has been widely used in allergen analysis, but it often requires long period of digestion which limits its application [163]. In this context, Qi et al., have applied a novel MNPs based hybrid material i.e., Try immobilized on hairy polymer-chain hybrid MNPs to shorten the digestion time and enhance the digestion efficiency [164]. Rapid digestion method based on as-prepared nanohybrid was used to detect milk allergens in baked food by ultrahigh-performance liquid chromatography-tandem mass spectrometry (UPLC-MS/MS). Due to this technique, immobilized Try was digested in a short period of time (15 min), with higher or equal sequence coverage compared to conventional free trypsin, which required 12–16 h for digestion. Furthermore, Try immobilized MNPs have also been used for continuous hydrolysis of casein, which is the main protein in milk, and known to produce several bioactive peptides after hydrolysis [98]. Atacan et al., covalently immobilized Try on tannic acid (TA) coated Fe_3_O_4_ MNPs to investigate the digestion of casein from bovine milk [165]. Digestion efficiency of casein was investigated using liquid chromatography–mass spectrometry (LC–MS/MS) technique, which confirmed the efficient digestion of casein by immobilized Try compared to free Try due to prevention of autohydrolysis.

## Conclusive remarks, challenges, and future directions

This review examines the prospects of surface-coated/functionalized magnetic nanostructured materials and their derived nanocomposites as exciting support candidates for the immobilization of various enzymes. It was observed that the surface coating/functionalization by inorganic materials (carbon, silicon groups, metal and metal oxides) and organic molecules (surfactants, small molecules, polymers, MOFs) can enhance the stability, and biocompatibility of MNPs, which consequently magnifies the application of these nanostructures for effective immobilization of enzymes. Although extensive research progress has been dedicated to the surface engineering of magnetic nanoparticles, a series of challenges still need to be addressed. For instance, a precise control over the size distribution and surface-coated shape of MNPs must be considered in future studies. Moreover, it is also important to address the issue of long-term stability of functionalized MNPs. Considering the future perspective, most of the applications, in particular, clinical aspects are still in the hypothetical phase necessitating consistent research investigations from multidisciplinary areas to realize its practical applications. Therefore, in addition to optimize the fabrication routes to synthesize magnetic nanostructures with better properties, the development of efficient, environmentally-friendlier, and stable surface-modification are also important. We envision that with sustained development, fabrication, and surface engineering strategies, numerous possibilities will arise to construct more and more multifunctional nanomaterials in the future to design immobilized biocatalytic systems for expanding the real-time application scope. Moreover, enzyme coated MNPs using the concepts of nanobiocatalysis also offer great potential in the field of biomedical applications including bioelectronics, bioconversion, and proteomics.

**Compliance with ethics requirements**

This article does not contain any studies with human or animal subjects.

## CRediT authorship contribution statement

**Muhammad Bilal:** Conceptualization, Data curation, Methodology, Writing – original draft, Writing – review & editing. **Hafiz M.N. Iqbal:** Conceptualization, Methodology, Writing – original draft, Writing – review & editing. **Syed Farooq Adil:** Methodology, Writing – original draft, Writing – review & editing. **Mohammed Rafi Shaik:**Methodology, Writing – original draft, Writing – review & editing. **Abdelatty Abdelgawad:** Data curation. **Mohammad Rafe Hatshan:** Funding acquisition, Data curation. **Mujeeb Khan:** Data curation, Methodology, Writing – original draft, Writing – review & editing.

## Declaration of Competing Interest

The authors declare that they have no known competing financial interests or personal relationships that could have appeared to influence the work reported in this paper.
